# Views on HPV-vaccination held by parents of Turkish and Moroccan origin in the Netherlands: an exploratory study using Q-methodology

**DOI:** 10.1186/s12889-026-26241-7

**Published:** 2026-01-17

**Authors:** Enise Çayci, Thijs van den Broek, Anna P. Nieboer

**Affiliations:** Department of Socio-Medical Sciences, Erasmus School of Health Policy & Management, Erasmus University Rotterdam, Rotterdam, The Netherlands

**Keywords:** HPV, Vaccine hesitancy, Gender, Q methodology, Ethnic minorities

## Abstract

**Background:**

Health disparities between people with and without a migration background remain a persistent issue across Europe, with individuals from non-European immigrant backgrounds often experiencing particularly negative outcomes. These populations also face higher risks of infection-related cancers, including those caused by Human Papillomavirus (HPV). Vaccination programs have the potential to mitigate such disparities. In the Netherlands, this potential is undermined by the low uptake among certain groups, especially individuals of Turkish and Moroccan origin, who represent two of the largest non-European ethnic minorities in the country. The underlying reasons for this low uptake are rarely explored in depth. This study, therefore, focuses on parents of Turkish and Moroccan origin living in the Netherlands, exploring their perspectives on HPV vaccination for their children aged 7 to 9 years. Building on the Health Belief Model, the study also considers how potentially gendered norms and expectations may influence parents’ attitudes toward vaccinating sons versus daughters.

**Methods:**

Q-methodology, a research technique that combines qualitative and quantitative methods to systematically study people's subjective viewpoints, was used to gain insights into parents' perspectives on HPV vaccination. 29 parents of Turkish or Moroccan origin in the Netherlands with children aged 7–9 were asked to rank 30 statements based on the Health Belief Model between April 2024 and January 2025. After ranking the statements, respondents were asked to explain the reasoning behind their rankings. By-factor analysis was used to identify distinct perspectives. The qualitative material was used to verify and refine the interpretation of the perspectives.

**Results:**

Three distinct perspectives were identified: 1. “Distrust, Uncertainty and Negative experiences as Barriers to Vaccination”, 2. HPV-Specific Vaccine Hesitancy: Concerns About Early-Age Vaccination and Necessity, and 3. Vaccination as key to preventing HPV-related disease.

**Conclusion:**

This study highlights the diversity of parental perspectives on HPV vaccination within Turkish and Moroccan communities in the Netherlands. While some parents support HPV vaccination as a preventive measure, others express great hesitancy. The root of this hesitancy differs somewhat between subgroups of vaccine-hesitant parents of Turkish and Moroccan origin. These findings underscore the need for tailored communication strategies to support informed decision-making and improve vaccine uptake.

**Supplementary Information:**

The online version contains supplementary material available at 10.1186/s12889-026-26241-7.

## Background

Non-European immigrants and their descendants often experience poorer health outcomes than native Europeans without an immigrant background [18, 31, 35, 67, 69]. Health inequalities between these groups are evident in both chronic conditions and infectious diseases [64]. Related to the latter, the risk of infection-associated cancers is particularly high for individuals with a non-European immigrant background compared to European natives [4, 5].

Human Papillomavirus (HPV) infections, the most prevalent viral infection of the reproductive tract, can result in various forms of cancer in both women and men [20]. HPV infection rates are higher among immigrants than among European natives without an immigrant background [10]. Furthermore, HPV-related cancer rates vary significantly amongst immigrant groups [17]. Preventive immunization against HPV can contribute to a reduction in the incidence of HPV-related cancers and precancerous lesions [9], and, consequently, mitigate inequalities in such health conditions [71].

Since 2007, European countries have implemented vaccination programs to prevent HPV-related cancers [14]. Initially, only girls were vaccinated, but it has become increasingly recognized that vaccinating boys is also crucial to reduce HPV transmission rates [59]. In response, many European countries have extended their vaccination programs to include boys in recent years [14]. The World Health Organization (WHO) has emphasized the importance of vaccination programs in addressing health inequities [71]. However, low HPV-vaccination uptake among people with a non-European immigrant background [16, 61] undermines the potential of these programs to reduce ethnic disparities in HPV-related cancers across Europe.

Unfortunately, the reasons underlying the lower HPV vaccination uptake among people with a non-European background remain largely unclear, as only few studies went beyond merely describing ethnic differences in HPV vaccination uptake [8, 37, 58]. The current study aims to fill this gap by exploring the multidimensional stances towards HPV vaccination of their children held by parents of Turkish and Moroccan origin in the Netherlands, as HPV vaccination rates are notoriously low for children of these parents [16]. For example, uptake in the four largest Dutch cities (Amsterdam, The Hague, Utrecht and Rotterdam) is 13% for girls of Turkish origin and 7% for girls of Moroccan origin [60]. Previous research has identified multiple potential drivers of vaccine hesitancy in ethnic minority populations, including limited knowledge about HPV and its vaccine, concerns about vaccine safety and side effects, institutional mistrust and perceived low susceptibility to infection (e.g., [3, 24, 36, 58]). Yet, little is known about the various ways in how constellations of such drivers may cluster together in minority populations with low vaccination rates. This is unfortunate, because people’s stance toward HPV vaccination entails multiple dimensions that jointly, rather than individually, shape the inclination to vaccinate (cf. [51]).

To frame our holistic exploration of parents’ perspectives on HPV-vaccination, we apply the Health Belief Model, a model which has been used as a heuristic framework in various vaccination studies focusing on ethnic minorities (e.g., [32, 49, 58]). As explained in further detail later, the current study moreover acknowledges that considerations about vaccination of boys and of girls may differ. To explore potential gendered stances on HPV vaccination among parents of Turkish and Moroccan descent, we conducted a Q-methodological study with 29 participants. This method examines subjective viewpoints by identifying individuals who rank statements similarly, indicating a shared perspective—in this case, on HPV vaccination.

### The Dutch context

This study is set in the Netherlands, where approximately 14.5% of the population has a non-European immigrant background, meaning that either they were born outside Europe or have at least one parent born outside Europe [13]. Among these individuals, people of Turkish and Moroccan origin are the two largest non-European ethnic groups [13]. The Netherlands faces significant disparities in HPV vaccination uptake among its citizens. Although the Dutch National Immunization Program (NIP) offers the HPV vaccine free of charge to both boys (as of 2022) and girls at the age of 10 (as of 2010) [53], HPV vaccination rates among Dutch individuals of Turkish and Moroccan origin remain significantly lower compared to other ethnic groups, including native Dutch individuals. In the Netherlands’ four largest cities (Amsterdam, Rotterdam, The Hague, Utrecht), the HPV vaccination uptake rate was 45% among native Dutch girls without a migration background of 11 years old, while vaccination rates among their counterparts of Turkish (13%) and Moroccan origin (7%) lagged behind substantially [60].

Our study sample includes both first- and second-generation parents. While the current study does not examine differences in attitudes by generational status, prior research suggests that generational status may influence system knowledge of preventive health services such as cancer screenings [6]. Including both generations allowed us to capture a broader range of perspectives on HPV vaccination.

### Health belief model

Prior work on the low HPV-vaccination uptake among people with a non-European migration background has mainly been descriptive, and only few studies have explored potential reasons for non-vaccination. In the current study, we explore the attitudes towards HPV vaccination held by parents of Turkish and Moroccan origin, because these attitudes plausibly shape their inclination to vaccinate their children. We will use the health belief model (HBM) as the central heuristic framework to help us understand people’s stances towards HPV-vaccination. The HBM was originally developed in the 1950 s to predict preventive health behavior and has since been applied across various disease contexts [56, 57]. Numerous studies have applied the HBM to vaccination behavior (e.g., [21, 30, 37]). Moreover, it has also been the main framework of multiple studies focusing on vaccination behavior in ethnic minorities (e.g., [32, 49, 58].

The HBM includes five key elements that are postulated to collectively explain the likelihood that an individual will engage in given health behavior to avoid undesirable health outcome [25]. For this study, we only apply the four elements that reflect internal beliefs, as we aim to examine the attitudes parents hold towards HPV vaccination. These elements are:

1. *Perceived susceptibility:* The individual’s belief about the risk of developing the health condition of interest, such as an HPV infection.
2. *Perceived severity:* The individual’s perception of the seriousness of the consequences associated with the condition of interest, such as the danger it might pose to health.
3. *Perceived benefits:* The anticipated advantages of engaging in the health behavior of interest, including its effectiveness in preventing the undesired outcome. In our case, protection against multiple types of cancer.
4. *Perceived barriers:* Potential obstacles to engaging in the health behavior of interest. These could vary from lack of knowledge of HPV to fear of harmful side effects.

Although we do not consider cues to action—the fifth HBM element; defined as external prompts that trigger the decision-making process—as part of our main analyses, we acknowledge that this is also a core element of the HBM. We will elaborate on what promising cues to action could be for people with specific stances toward HPV vaccination, as this may inform the development of more effective health communication strategies.

### The role of gender

In the current study, we go beyond the straightforward application of the HBM model to HPV-vaccination by applying a gender perspective to it. Although the HBM model considers both ethnicity and gender as individual determinants of health beliefs, attention for intersections between ethnicity and gender, which some public health scholars have long called for [40], has hitherto been limited in research on HPV-vaccination. This is unfortunate, because there are reasons to suspect that the beliefs of people of Turkish and Moroccan origin about HPV-vaccination of daughters and of sons, respectively, may differ notably in some respects.

A factor potentially influencing gender differences is the role of religious and cultural norms. Individuals of Turkish and Moroccan descent are often part of Muslim communities with strong norms emphasizing premarital abstinence. Premarital sexual relationships are often perceived as morally unacceptable by individuals of Moroccan and Turkish descent [33], aligning with Islamic teachings that classify such behavior as *zinah* (illegitimate sexuality). Illegitimate sexuality is considered both religiously prohibited and socially undesirable, applying to both men and women [48]. However, an in-depth analysis by Smerecnik et al. [63] identified the existence of a (hetero)sexual double standard ((H)SDS) regarding sexual activity, indicating that Muslim men do not always adhere to this rule, despite considering premarital sexual activity *haram* (illicit). Similarly, Buunk and Dijkstra (2017) suggest that Moroccan and Turkish men are more likely than women to engage in premarital sexual relationships. This may lead to the perception that girls are less susceptible to HPV, as they are not expected to be at risk of sexually transmitted infections [7, 24, 58].

Furthermore, concerns about sexual activity may influence HPV vaccination decisions. Some parents fear that HPV vaccination might encourage sexual activity [7, 24, 58]. In HBM terms, this could be seen as a perceived barrier to vaccination. Consistent with the (H)SDS described above, research suggests that premarital sex among Muslim men is less scrutinized, whereas Muslim women’s sexuality tends to be more strictly controlled [22]. Hence, concerns that HPV vaccination may encourage sexual activity may be a stronger barrier to vaccination of girls than to vaccination of boys.

Additionally, perceptions regarding severity of HPV for girls and for boys may differ. While awareness of HPV’s link to cervical cancer is relatively high, knowledge of its association with penile and other cancers remains limited [45]. As a result, there is a widespread misconception that HPV is solely a women’s health issue [52], indicating that parents might not always perceive its threat to both sons and daughters. These examples illustrate that a gender perspective, which recognizes that parents' considerations about the vaccination of sons and daughters may differ, is essential when applying the HBM to Turkish and Moroccan origin parents’ stances towards HPV vaccination.

## Methods

### Study design

This study employed Q-methodology, an exploratory technique designed to probe subjective viewpoints by identifying individuals who have similarly ranked statements, suggesting a shared perspective on a particular topic [70]. We specifically focused on identifying the varying attitudes among parents of Turkish and Moroccan descent in the Netherlands. Participants ranked statements about HPV vaccination to reveal subjective viewpoints on the matter. Additionally, semi-structured qualitative interviews were conducted to explore participants' rationale for their rankings.

We performed by-person factor analysis to identify significant correlations among the placement of statements [70]. Each resulting factor represented a distinct viewpoint on the matter based on the pattern of statements that belongs to the factor. This pattern of statements served as the foundation for interpreting and describing each viewpoint. Moreover, the qualitative interview data was used as supporting material for the interpretation of each viewpoint.

### Participants

We recruited parents who had children aged 7–9 as participants for the current study, as children aged 10 are invited for their first HPV vaccine in the Netherlands. Our study specifically focused on parents of Turkish and Moroccan origin residing in the Netherlands. Therefore, another inclusion criterion was that the participants identified as being of Turkish and/or Moroccan origin but were living in the Netherlands. Parents were recruited between April 2024 and January 2025.

Initially, respondents were purposively recruited using the informal networks of the researchers involved. To further expand our recruitment efforts, we recruited via social media and visited community centers. Furthermore, respondents were also recruited using the snowball method [38], by asking participating respondents if they knew other people meeting the inclusion criteria. Additionally, a small number of respondents were recruited purposively on the street, meaning that they were approached and briefly screened to determine if they met the study’s inclusion criteria. Each participant received a €20 gift card as an expression of gratitude for their participation.

These efforts resulted in a sample of 29 respondents (17 parents of Turkish origin and 12 parents of Moroccan origin), which is quite common in Q-studies [19, 70]. Of these, 19 were female and 10 were male. The majority of parents had obtained higher education (HBO or WO i.e., professional or academic degrees). The median age category was 35–44. An overview of participants’ background characteristics is presented in Table 1. More detailed information can be found in the Supplementary Materials. To enhance narrative clarity and readability in the presentation of interview excerpts, pseudonyms were assigned to participants. These names were selected to be culturally plausible but deliberately non-stereotypical and do not correspond to participants’ actual names.

**Table 1:** A Summary of the Background Characteristics of the Participants (*n* = 29)

Pseudonym	Gender	Age category	Ethnic Origin (generation)	Child(ren)’s gender	Total # of children	Education
Aylin	F	35–44	Turkish (II)	F & M	2	Higher education
Burcu	F	35–44	Turkish (II)	F & M	2	Higher education
Kenan	M	25–34	Turkish (I)	F	2	Secondary & Vocational education
Canan	F	45–54	Turkish (I)	M	3	Secondary & Vocational education
Ozan	M	25–34	Turkish (II)	F	2	Secondary & Vocational education
Elif	F	35–44	Turkish (II)	F	2	Higher education
Karim	M	35–44	Moroccan (I)	F	3	Secondary & Vocational education
Selin	F	45–54	Turkish (I)	F	2	Secondary & Vocational education
Imane	F	35–44	Moroccan (II)	F	2	Secondary & Vocational education
Leyla	F	35–44	Turkish (II)	F	3	Secondary & Vocational education
Yasemin	F	35–44	Turkish (I)	F	3	Higher education
Sara	F	35–44	Moroccan (II)	M	3	Higher education
Rania	F	45–54	Moroccan (I)	F	2	Higher education
Nadia	F	25–34	Moroccan (I)	F	2	Secondary & Vocational education
Samira	F	35–44	Moroccan (I)	F	3	Primary & Preparatory education
Ela	F	25–34	Turkish (I)	M	2	Secondary & Vocational education
Idil	F	35–44	Turkish (II)	F	3	Higher education
Deniz	M	25–34	Turkish (II)	F & M	5	Higher education
Eren	M	35–44	Turkish (II)	F	2	Secondary & Vocational education
Amal	F	35–44	Moroccan (II)	M	3	Secondary & Vocational education
Salma	F	35–44	Moroccan (II)	F	3	Higher education
Zahra	F	45–54	Moroccan (I)	F	2	Primary & Preparatory education
Murat	M	35–44	Turkish (I)	F	2	Secondary & Vocational education
Amira	F	35–44	Moroccan (II)	F	3	Higher education
Adam	M	35–44	Moroccan (I)	F	3	Secondary & Vocational education
Mert	M	35–44	Turkish (I)	F	1	Higher education
Ata	M	35–44	Turkish (I)	F	1	Higher education
Naima	F	25–34	Moroccan (II)	F	4	Higher education
Baran	M	25–34	Turkish (II)	M	1	Secondary & Vocational education

### Statement set development

As described above, the HBM posits that four belief aspects jointly constitute people’s stance towards HPV-vaccination: perceived susceptibility to HPV infections, perceived severity of HPV, perceived benefits of HPV vaccination and perceived barriers to HPV vaccination. We therefore first conducted a review of scientific, popular, and grey literature to compile a list of potentially relevant perceptions on HPV vaccination of each of these four dimensions. Based on this review, one researcher (EC) prepared an initial set of 73 statements. This set was then reviewed by two other researchers (TB, AN); some statements were merged or removed due to redundancy, and the wording was adjusted to a B1 Dutch language proficiency level. To cover the gender perspective in the set of statements, separate statements referring specifically to boys and girls were included for selected aspects as a means of exploring whether parents’ considerations regarding the vaccination of sons and of daughters differ. The resulting set of 30 statements was then reviewed by two additional readers. Both readers have extensive expertise with either conducting a Q-study in people of Turkish and Moroccan origin or in prevention of infectious diseases. Neither additional reader found it necessary to propose further modifications. The final set of statements was translated into Turkish by the principal investigator and to Arabic by an Arabic-speaking fellow researcher, who was also involved in reviewing the statements. To assess the applicability and comprehensibility of the statement set, a pilot study involving two respondents was conducted first. Based on the results, we concluded that the set of statements was complete and comprehensible for the population of interest. The final set of 30 statements, along with their distribution in the factor arrays for the extracted perspectives, is presented in Table 3. The complete English, Dutch, Turkish and Arabic translations are provided in the Supplementary Material 3.

### Procedure

The session followed a structured format: First, the respondent received a brief introduction to the study supplemented by verbal clarification as needed. Next, participants completed an 8-item questionnaire covering demographic information about gender, age, child’s gender, child’s age, origin, generation of migration, educational level and employment status. The questionnaire is provided in Supplementary Materials 1. Each participant then received 30 statements printed on cards and presented in a random order. They were asked to carefully read each card and request clarification if needed. Then, participants were instructed to sort the statement cards into three piles: a first pile for statements they agreed with, a second pile for statements they disagreed with, and a third pile for statements they felt neutral about or did not know.

**Fig. 1:**
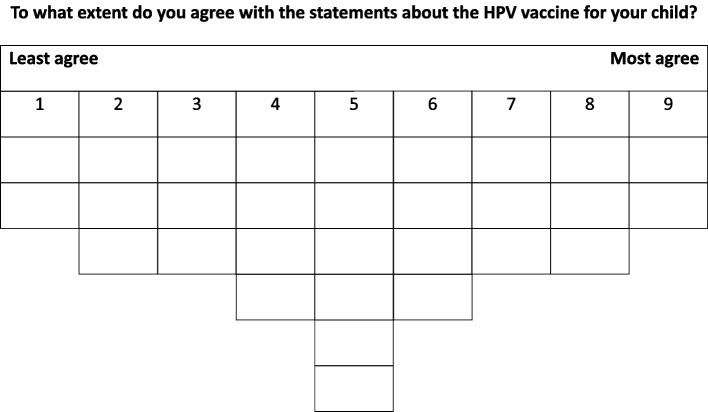
Scoresheet for ranking agreement statements

In the next step, participants were asked to re-read all the cards, starting with the pile of statements they agreed with. Each card was then ranked on a score sheet such as presented in Fig. 1 according to the participant's level of agreement. The two cards they agreed most with were placed in the rightmost column. This process was repeated for the pile containing the statements they disagreed with, with the two cards they least agreed being placed in the leftmost column. Statements considered neutral were placed in the remaining spaces in the center of the score sheet. The further the column was positioned to the right, the more the participants agreed with the corresponding statement. Participants were allowed to change the positioning of the cards during this procedure. After arranging the statements, each respondent was prompted to explain the reasoning behind their rankings for statements. These explanations took the form of a semi-structured interview in which participants explained their reasoning behind the placement of the statements. The entire procedure, including both ranking and participants’ explanations, was audio-recorded with their informed consent.

All sessions were conducted face-to-face by the principal investigator at the participants’ preferred locations, in their preferred language. Dutch was chosen as the principal language in 20 interviews, whereas 4 respondents chose to do the interview completely in Turkish. One interview was partially conducted in Spanish, using the Arabic translation of the cards while explanations were provided in both Dutch and Spanish. For two respondents with limited Dutch proficiency, an informal translator assisted, providing live Berber translations. Participants occasionally code-switched i.e., alternating between languages within a single interview, enriching expression. As the PI is fluent in Dutch, Turkish and Spanish, the languages of these interviews did not affect the trustworthiness of interpretation. Using participants’ preferred languages and code-switching also fostered trust. Interviews were transcribed verbatim using Amberscript, whereby in-situ translations were used as data for the interviews partially conducted with the help of informal translators. Field notes were taken throughout and after the sessions to capture contextual information.

### Data analysis

The individual rankings of the 29 participants underwent by-person factor analysis, specifically centroid extraction followed by varimax rotation, using the qfactor command in Stata [1]. This was done to identify shared viewpoints on HPV vaccination among those rankings. Furthermore, the analysis distinguished between consensus statements (statements with consistent rankings across different factors) and differentiating statements (statements with significantly differing rankings from other factors).

We performed a Scree test to determine the number of perspectives [12]. This involves plotting the eigenvalues to visually identify where the curve levels off, which indicates the optimal number of distinct perspectives to retain. A factor loading threshold of ± 0.36 (*p* < 0.05) was used to identify which participants significantly aligned with each factor, and only factors with at least three significantly loading Q-sorts were retained. The resulting perspectives were considered representing different viewpoints towards HPV vaccination based on the set of statements.

The rotated factor loadings for individual participants, indicating how strongly each participant’s responses aligned with each extracted perspective are provided in Table S4 (Supplementary Material). Qualitative interview data from respondents were used to validate and clarify interpretations of the perspectives they aligned with. Specifically, for each perspective, we selected quotes from the five highest aligning participants on the statements they most agreed with (scores 8–9) and the statements they least agreed with (scores 1–2). These quotes were used to systematically form the foundation of the narrative for each perspective, while additional quotes from other participants and other statements were included to further support and illustrate the interpretation of the factors.

## Results

Twenty-nine participants completed the ranking (median age category, 35–44; 59% Turkish, 62% women; 72% has at least one daughter; Table 1). The analysis resulted in three distinct perspectives, that together explained 57.9% of the variance in the data, with twenty-eight participants loading significantly on one of the perspectives (*p* < 0.05). An overview of the key characteristics of each perspective is presented in Table 2. The factor arrays of the perspectives are presented in Table 3. A factor array represents a ranking of statements that reflect the viewpoint of each identified perspective, based on the factor analysis results.

**Table 2:** Summary of the key characteristics of each perspective on HPV vaccination

Perspective	Eigenvalue	Explained variance	Participants loading to perspective (N)	Gender (F/M)	Ethnic origin (Turkish/Moroccan)	Summary of findings
1: Distrust, Uncertainty and Negative Experiences as Barriers to Vaccination	7.62	39.49%	12	7/5	5/7	Parents are generally hesitant towards HPV vaccination due to a combination of general distrust in vaccines and authorities, limited knowledge about HPV and negative experiences with immunization. They emphasize children’s autonomy in decision-making and do not perceive social, cultural or religious barriers
2: HPV-specific Vaccine Hesitancy: Concerns abour Early-age Vaccination and Doubts about Necessity	1.84	21.58%	6	4/2	4/2	Parents’ hesitancy is specific to HPV vaccination rather than vaccines in general. They are concerned about vaccinating children at a young age, the need to explain the vaccine’s necessity, and potential side effects. Decision-making is viewed primarily as a parental responsibility, and cultural concerns relate to discussing sexuality, not the vaccine itself
3: Vaccination as Key to Preventing HPV-related Disease	6.05	38.92%	10	7/3	7/3	Parents support HPV vaccination, emphasizing its role in cancer prevention and overall health protection. They value immunization and recognize risks for both boys and girls. Parents perceive the vaccine as compatible with cultural and religious beliefs and view vaccination as a responsible, proactive health decision for their children

**Table 3:** Factor arrays representing three perspectives on HPV-vaccination

Statement		Perspective* (factor)		
		1	2	3
*Perceived susceptibility*				
1	The risk of girls contracting HPV is small	6	4	4
2	The risk of boys contracting HPV is small.^a^	5	5	4
3	Girls cannot get HPV because they do not engage in sexual activity before marriage	5	3	2
4	Boys cannot get HPV because they do not engage in sexual activity before marriage	5	4	2
5	Vaccination against HPV is only necessary when people become sexually active.^a^	4	5	5
6	Illness and health are in God's hands, so I do not find the HPV vaccine necessary	6	5	1
7	It is too early to vaccinate girls against HPV at the age of 10	7	9	6
8	It is too early to vaccinate boys against HPV at the age of 10	7	9	6
9	Boys and girls can infect each other with HPV, so both should be vaccinated	5	7	9
*Perceived severity*				
10	I do not understand why I should vaccinate my child against HPV	6	6	4
11	Vaccination against HPV is important to prevent cancer	5	2	9
12	HPV is a danger to the health of boys.^a^	5	6	5
13	HPV is a danger to the health of girls.^a^	7	8	7
*Perceived benefits*				
14	The HPV vaccine protects my child against various types of cancer	3	4	8
15	Getting vaccinated against HPV has more benefits than drawbacks	4	2	8
16	I am not vaccinated against HPV myself, so my child does not need it either	4	8	4
17	The HPV vaccine aligns with my faith because I believe I should take good care of my health	3	5	6
*Perceived barriers*				
18	I am against vaccinations in general	9	3	2
19	The HPV vaccine has harmful side effects for my child	8	7	6
20	I do not know what the possible side effects of the HPV vaccine are for my child	8	5	7
21	I do not know enough about the HPV vaccine to decide whether to vaccinate my child	8	6	5
22	There are substances in the HPV vaccine that are haram	4	5	**1**
23	Vaccinating my child against HPV would harm my family's honour	1	1	3
24	I trust the government to ensure that the HPV vaccine is safe	2	3	8
25	I trust manufacturers to make a safe HPV vaccine	1	1	7
26	Vaccinating my child against HPV encourages sexual behaviour	2	7	3
27	My child receives too many vaccinations at that age	6	8	5
28	It is a hassle to have my child vaccinated twice a year	3	2	5
29	Children should decide for themselves about the HPV vaccine when they are old enough	9	6	5
30	The HPV vaccine goes against my culture	2	4	3

^*^Perspective: 1, “Distrust, Uncertainty and Negative Experiences as Barriers to Vaccination”; 2, “HPV-specific Vaccine Hesitancy: Concerns about Early-Age Vaccination and Doubts about Necessity”; 3, “Vaccination as Key to Preventing HPV-related Disease”. ^a^Consensus statement

The values in each column represent the scores assigned to each statement in the factor array for the corresponding perspective. Higher numbers indicate statements that were more strongly agreed with in that perspective

### Perspective 1: “Distrust, Uncertainty and Negative Experiences as Barriers to Vaccination”

Perspective 1 has an eigenvalue of 7.62 and accounts for 39.49% of the study’s explained variance. Twelve participants are significantly associated with this perspective, of whom seven are female and five are male. Five participants are of Turkish origin and seven are of Moroccan origin. Among the loading participants, five were first-generation and seven were second-generation parents.

This perspective reflects great hesitancy towards HPV-vaccination, which is embedded in a highly negative attitude towards vaccination in general. This negative attitude is driven by three interconnected barriers: (1) general distrust towards immunization, (2) insufficient knowledge about HPV, and (3) negative experiences with immunization in others. Each barrier influences the other, ultimately reinforcing a negative attitude towards HPV vaccination.

The general distrust towards immunization (statement 18 ranked 9) is primarily linked to a lack of trust in manufacturers (st. 25: 1) and the government (st. 24: 2). A major contributor to this issue, as mentioned by respondents, was the COVID-19 pandemic. Several parents aligning with this viewpoint highlighted that either the government’s handling of the situation during COVID-19 or stories about people getting sick after a COVID-19 vaccination made them lose trust in both entities.

Amal (F, 35–44 y.o., Moroccan (II), 3 children) states: *“I don’t trust manufacturers. I do trust the government a bit more, one step further. But it’s just because of what’s been happening in the world in recent years. I’m not a conspiracy theorist, but sometimes I see conspiracies that make me think there’s something to it, and I think this is just one of them. Just look at the COVID-19 vaccine. It was already said at the time that it was way too short a time to administer it. But yeah, where was the government then? And where was the manufacturer? The manufacturer only made millions or billions from it and didn’t give anything back to the world.”*

Alleged negative experiences of people in their surroundings are also cited as reasons for not vaccinating children, reinforcing the belief that the HPV vaccine has harmful side effects (st. 19: 8). Sara (F, 35–44 y.o., Moroccan (II), 3 children) mentions: *“It’s just very difficult, those side effects, especially harmful side effects. And also, because I know someone who got the HPV vaccine at a young age and developed diabetes as a result. I don’t know if there’s really a causal link, but that’s what is thought. […] I think those vaccines can cause a lot of harm, of which we don’t know much.”*

The fear that the HPV vaccine may have harmful side effects is intertwined with uncertainty about potential side effects for their children (st. 20: 8) *[“I don’t know what’s in it, so I’d rather not do it, also considering all those negative experiences associated with it.”* – Idil (F, 35–44 y.o., Turkish (II), 3 children)]. Participants with this perspective fear that side effects of the HPV vaccine may be harmful, because the potential side effects are unknown to them.

What emerges is that parents frequently reported feeling they possessed insufficient knowledge about the HPV vaccine (st. 21: 8) and expressed doubts regarding its purpose, namely protection against various types of cancer (st. 14: 3). These perceptions were often associated with uncertainty about the value of vaccinating their children against HPV (st. 10: 6). While parents generally acknowledged that HPV could have severe health implications, they tended to perceive it as a greater danger to the health of girls (st. 13: 7) than to the health of boys (st. 12: 5).

Something this perspective prioritizes is the bodily autonomy of children, as parents highly agree that children should decide for themselves about the HPV vaccine when they are old enough (st. 29: 9). The definition of ‘old enough’ varies across respondents as some consider it to be at legal age of 18, whereas others would let their children decide when they are around “*16,17 years old*” [- Idil (F, 35–44 y.o., Turkish (II), 3 children)]. They agree, however, that, for girls (st. 7: 7) as well as for boys (st. 8: 7), the age of 10 is too young to be vaccinated against HPV.

Furthermore, they do not tend to agree with the statement that the HPV vaccine could damage a family's honour (st. 23: 1), nor do they believe it conflicts with their culture (st. 30: 2). Several parents acknowledge their Muslim background during the interview and occasionally link their religion to their culture. However, none state that immunization -whether against HPV or in general- is contrary to their religious or cultural beliefs.

Amal (F, 35–44 y.o., Moroccan (II), 3 children) states: “*I can’t imagine that there is anything in my culture—or maybe I would link it more to my faith— has an opinion on this. Because even from a religious perspective, as a parent, you just have to do what is best for your child. And of course, we do have certain guidelines, but I can’t imagine anyone saying that you shouldn’t vaccinate because it wouldn’t align with our culture or faith or something like that.”*.

### Perspective 2: “HPV-Specific Vaccine Hesitancy: Concerns about Early-Age Vaccination and Doubts about Necessity***”***

Perspective 2 has an eigenvalue of 1.84 and accounts for 21.58% of the study’s explained variance. Six participants are significantly associated with this perspective of whom four are female and two are male. Four participants are of Turkish origin and two are of Moroccan origin. Among the loading participants, generational status is evenly distributed, with three first-generation and three second-generation parents.

Although this perspective shares some hesitancy toward HPV vaccination with Perspective 1, its concerns are more rooted in issues specific to HPV rather than general opposition to vaccination. These parents are generally less opposed to vaccination overall (st. 18: 3); however, their concerns are more narrowly focused on the HPV vaccine. A key concern relates to their perception of their children's susceptibility to HPV. They believe it is too early to vaccinate children against HPV (st. 7 & 8: 9). Several parents feel that children at this age are not, and should not be, sexually active. As a result, they believe children should neither be burdened with nor exposed to topics related to sexuality, including the HPV vaccine.

Aylin (F, 35–44 y.o., Turkish (II), 2 children) states: *“Children are really being forced at an early age to deal with sexuality. I find that truly bizarre. I mean, if you're 10, you're really not thinking about that yet. I think children should remain children, and at ten years old, you're still very much a child.”*

Others report difficulties in *explaining* the necessity of the vaccine to young children, fearing that such discussions may unintentionally lead to conversations about sensitive topics such as sexuality, which they believe is inappropriate at a young age. This concern is closely tied to cultural values and norms. While they do not perceive the HPV vaccine itself as conflicting with their cultural values (st. 30: 4), their hesitation stems from the timing of introducing discussions about sexual activity. These parents also fear that raising these topics too early could encourage sexual behaviour in their children (st. 26: 7). This perception appears more frequently among these parents than in other perspectives and serves as a notable barrier to HPV vaccination specifically.

Adam (M, 35–44 y.o., Moroccan (I), 3 children) states: *“I think vaccinating against HPV at a young age is too early. Because if you have to do this for a ten-year-old girl or a ten-year-old boy, you obviously have to explain why they need to get it. At the age of 10, it is difficult to properly explain why this is necessary. Especially in our culture, it is challenging—perhaps in other cultures, it is not a problem. But in our culture, it is difficult to explain why…”.*

Distrust in manufacturers of the vaccine, similar to Perspective 1, also negatively affects these parents' stance toward HPV vaccination (st. 25: 1). One contributing factor to this mistrust is, similarly to perspective 1, the COVID-19 pandemic. Another key issue is uncertainty about the composition of vaccines and their ingredients. Parents with Perspective 2 specifically believe that it is the responsibility of the pharmaceutical industry to provide accessible and transparent communication about vaccine contents, since these companies are the ones producing them. Consequently, this may explain why their mistrust is less directed toward the government (st. 24: 3).

Amira (F, 35–44 y.o., Moroccan (II), 3 children) further elaborates: *"You don't know if it's safe. Look, maybe there is enough information about whether it's safe or not, but I don't always trust 100% what is considered safe because I don't know everything that is inside."*

Adding to this concern, Yasemin (F, 35–44, Turkish (I), 3 children) states: *“[…] When you buy any medicine, there is a leaflet inside. It's this long, but they finish it with just a tiny couple of lines at the bottom. I mean, side effects like vomiting, *etc*., are not written in a language I can understand. You produce that medicine, you write it in your own language, but you do not inform me."*.

Another concern parents with this viewpoint specifically face is that children – according to their parents – already receive multiple vaccinations around the age of 10 (st. 27: 8). While they are less hesitant against ‘traditional’ vaccines that have long been part of immunization programs, they struggle to understand the necessity of newer vaccines, such as the HPV vaccine (st. 10: 6). They often compare the present to the past, noting that such vaccines did not exist before, yet people seemed to manage without them (st. 16: 8). Accordingly, these parents are not convinced of the benefits of HPV-vaccination; they do not perceive HPV vaccination as beneficial (st. 15: 2) and do not believe it prevents cancer (st. 11: 2 & st. 14: 4).

Amira (F, 35–44 y.o., Moroccan (II), 3 children) states:* “What has changed in terms of diseases then and now? I understand that times have changed and that youth has changed, but, I was also vaccinated against everything back then. I did that too, and this is, so to speak, something new. Why was it not as significant back then as it is now? …”.*

Although these parents struggle to understand the necessity of the HPV vaccine, they do acknowledge the severity of HPV-related disease, as they believe HPV poses a health risk to boys and, particularly, girls (st. 12: 6; st. 13: 8). At the same time, parents in perspective 2 report a moderate sense of being sufficiently informed to make a decision about HPV vaccination, including awareness about its potential side effects (st. 21: 6; st. 20: 5). This contrasts with perspective 1, where parents generally feel insufficiently informed to decide about HPV-vaccination for their children (st. 21: 8). Despite feeling moderately well-informed, parents in perspective 2 express strong concerns about the HPV vaccine’s safety, believing it could cause harmful side effects in their children (st. 19: 7). This combination suggests that their hesitancy is HPV-specific, driven not by a lack of information in general but by concerns about HPV vaccine safety.

In contrast to parents in Perspective 1, these parents do not specifically believe it is necessary to let children decide about the HPV vaccine when they are older (st. 29: 6), indicating that they see HPV vaccination primarily as a parental responsibility. This emphasis on parental control is accompanied by a general reluctance toward HPV-vaccination. Importantly, their focus on making the decision themselves does not reflect social pressure. Similarly to parents in Perspective 1, they do not report experiencing social pressure to refrain from vaccination in order to preserve family honour (st. 23: 1).

### Perspective 3:* “*Vaccination as Key to Preventing HPV-related Disease*”*

Perspective 3 has an eigenvalue of 6.05 and accounts for 38.92% of the study’s explained variance. Ten participants are significantly associated with this perspective of whom seven are female and three are male. Seven participants are of Turkish origin and three are of Moroccan origin. Among the loading participants, six were first-generation and four were second-generation parents.

This perspective reflects a positive attitude toward HPV-vaccination, driven by the perceived benefits, particularly its role in cancer prevention (st. 11: 9). Parents with this viewpoint generally support immunization (st. 18: 2), believing it to be essential in protecting against serious diseases.

Burcu (F, 35–44 y.o., Turkish (II), 2 children) states: *“I am definitely in favor of vaccinations, because thanks to those vaccinations, all those diseases have been eliminated…”.* Ata (M, 35–44 y.o., Turkish (I), 1 child) adds: *“I think all vaccines have important features because, thanks to vaccines, very serious and widespread diseases have been prevented. After all, a vaccine is there to protect against a disease.”*

They recognize that immunization prevents HPV-related cancers (st. 11: 9; st. 14: 8). It is their belief that the benefits of the HPV vaccine outweigh its disadvantages (st. 15: 8). Ata (M, 35–44 y.o., Turkish (I), 1 child) states: *“A vaccine has been developed to prevent cancer, and since this is a proven matter and cancer is one of the most significant diseases of our time, I would be most in favor of it.”.*

Similar to the first and second perspectives, these parents see HPV as greater danger for girls (st. 13: 7) than for boys (st. 12: 5). Yet, they believe that boys and girls should both be vaccinated, because boys and girls can transmit HPV to each other (st. 9: 9). They acknowledge that girls and boys are both at risk of HPV infection (st. 1 & 2: 4) and believe it would be incorrect to assume that boys (st. 4: 2) and girls (st. 3: 2) cannot contract HPV simply due to presumed abstinence from sexual activity before marriage. Some parents view it as naïve to believe that they can fully control when their children become sexually active, even if they would ideally prefer such control. Others emphasize that children possess autonomy and will ultimately make their own decisions as they grow older. Mert (M, 35–44 y.o., Turkish (I), 1 child) states: *“The age of sexual intercourse has also changed. I mean, the conditions… Both women and men are engaging in sexual activity before marriage. So, there is also a risk of transmission through this route.”*

Religious or cultural beliefs do not constitute a barrier to vaccination for these parents. They view health as a medical and scientific matter rather than as one influenced by culture (st. 30: 3). They firmly believe that one should not rely solely on fate but should take all possible measures to prevent illness before it occurs. This perspective is reflected in how they ranked the statement, "Illness and health are in God’s hands, so I do not find the HPV vaccine necessary" (st. 6: 1). For instance, one parent explains that while belief in fate is common, taking precautions remains essential: *“Okay, we all believe in fate, but of course, precautions need to be taken. If you don't take precautions and the disease spreads, saying ‘Oh well, it came from God’ seems a bit illogical to me. […] These things have already been explained scientifically. So, I don't think this matter is related to faith.” –* Mert (M, 35–44 y.o., Turkish (I), 1 child).

Another respondent seconds this view by emphasizing the religious principle of *Sebep*, which refers to the belief that while fate is determined by God, individuals are still responsible for taking necessary actions. This concept aligns with the idea that trusting in divine will does not mean neglecting one's duty to act. Consequently, this perspective explains why the HPV vaccine is seen as compatible with their faith, as they believe their faith prescribes that they should maintain good health (st. 17: 6). Naima (F, 25–34 y.o., Moroccan (II), 4 children) elaborates: *“From the belief that you should certainly take good care of yourself, but that there must also be something like ‘Sebep.’ And Sebep is… You have to take certain steps to get to where you want to be. You can't just sit and think, ‘Hey, the vaccination shot will come to me.’ No, you have to take certain steps in order to receive a vaccination shot.”*

These explanations highlight that for these parents, immunization is a proactive and rational decision based on scientific understanding and personal responsibility, which, in some cases, is further reinforced, rather than undermined by religious beliefs. They also do not believe the vaccine contains illicit substances (st. 22: 1). Hence, their perspective aligns with the idea that faith does not eliminate the need for precautionary measures, but rather that it can encourage action.

## Discussion

Although various studies, including a Q-study by Patty et al. [51], have explored parental beliefs about HPV immunization, this is the first study to explicitly focus on the perspectives of parents of Turkish and Moroccan origin in the Netherlands. These groups represent the largest non-European ethnic minorities in the country and have notoriously low HPV-vaccination uptake rates. Additionally, this study is the first to examine the potential gendered perspectives these parents may hold towards HPV-vaccination. Our study identified three distinct viewpoints on HPV immunization among parents of Turkish or Moroccan origin.

### Distrust, Uncertainty and Negative experiences as Barriers to Vaccination

Parents aligning with this viewpoint have a negative attitude towards vaccination due to three main barriers they perceive: (1) general distrust towards immunization; (2) insufficient knowledge about HPV, and (3) negative experiences with immunization of others. These findings align with previous research on the barriers parents across various ethnic backgrounds in Europe perceive towards HPV-vaccination [34, 42]. Our study indicates that the concerns expressed by the parents holding this viewpoint closely resemble those reported in other European contexts (e.g., [23, 34, 42]). Religious beliefs and cultural beliefs specific to parents of Turkish or Moroccan origin did not clearly emerge as influential in the decision-making about HPV-vaccination for their children among parents holding this perspective.

One of the matters these parents agree the most on is that children themselves should decide on HPV vaccination when they are old enough. This preference reflects recognition of their children’s agency and autonomy in health-related decision-making. At the same time, uncertainty surrounding the HPV vaccination, particularly regarding its potential side effects, appears to shape how parents evaluate risks. Although parents acknowledge that HPV poses a health risk to both boys and girls to some extent, uncertainty about the vaccine side effects evokes negative affective responses that weigh heavily in their risk evaluations. Research on risk perception suggests that such judgements are often guided less by analytical assessments of probability and benefit, and more by intuitive, affective responses to potential harm [62]. In this context, parents often choose not to vaccinate their children against HPV at this age, perceiving this as less distressing than actively choosing for vaccination, which is associated with feared and uncertain outcomes. Consequently, transferring the decision to their children allows parents to reconcile ongoing concerns about vaccine safety with an emphasis on the children’s autonomy and personal responsibility.

### HPV-Specific Vaccine Hesitancy: Concerns About Early-Age Vaccination and Doubts about Necessity

This viewpoint reflects a negative attitude towards HPV-vaccination, primarily due to its timing and the challenge of explaining its purpose to young children. These concerns are specific to HPV-vaccination rather than to immunization in general. They believe that the age of 10 is too young to understand the purpose of the vaccine. Earlier research documented similar concerns in other groups, showing that many parents prefer not to vaccinate their children against HPV at a young age [29, 55]. One reason for hesitation about the vaccine’s timing is that some parents do not understand why vaccinating pre-pubertal children before sexual debut is important. They may be unaware that early vaccination ensures immunity before potential HPV exposure, leading them to view vaccination at a young age as unnecessary or premature [29]. Additionally, the belief that children should not be informed about sexuality until they are older could reinforce their hesitation.

Another key concern is the fear that initiating a discussion about sexuality might inadvertently encourage sexual behaviour. Although existing literature does not suggest that parent–child discussions on sexual health leads to increased sexual activity [28], this fear persists among many parents regardless of ethnic, religious or cultural background [47]. This is also the case amongst some of the parents we spoke to. Cultural factors play a significant role in parent–child communication regarding sexual health. Not all parents are willing to openly discuss sexual health with their children, as it remains a taboo topic [47]. This idea reinforces the perception that framing HPV as a primarily sexually transmitted infection acts as a barrier to HPV vaccine uptake [11, 50].

### Vaccination as Key to Preventing HPV-related Disease

Parents with this viewpoint have a positive attitude towards HPV-vaccination, which stems from the perceived preventive benefits of HPV immunization, especially for cancer prevention. This element has also been noted among parents that support HPV-vaccination in prior studies [29, 42]. Interestingly, although these parents showed high willingness to vaccinate against HPV, they still perceive that they have insufficient knowledge on the potential side effects the vaccine might have. This is noteworthy as it suggests that while these parents acknowledge the health benefits of vaccination and are pro-actively willing to act against HPV, they remain cautious about the risks of the HPV vaccine, reflecting a commonly reported tension between perceived benefits and perceived risks of HPV-vaccination.

The concern of insufficient knowledge could be explained by the fact that parents in the Netherlands are known to have little knowledge of HPV compared to their counterparts in other European countries [42]. Although insufficient knowledge did not appear as a barrier to HPV-vaccination in the eyes of the parents holding this perspective, it still remains one of the largest barriers to HPV-vaccination [29, 34]. Therefore, it is important to inform parents adequately as knowledge on the vaccine is a broadly identified driver to HPV vaccine acceptance [41].

Another notable finding in this perspective is that these parents were the only ones who remained relatively neutral about the age of HPV vaccine administration, in contrast to other perspectives in our study, which viewed the vaccine as being administered too early. This could be explained by parental attitudes, as those who are more supportive of HPV-vaccination tend to be less concerned about pre-teen vaccination [43].

In terms of religious and cultural beliefs, these parents do not view their faith or culture as a barrier to HPV-vaccination. Some even highlight how Islamic beliefs reinforce their positive stance towards HPV-vaccination. This is in line with the idea that core Islamic teachings do not oppose vaccination, but in fact, can encourage it as a means of protecting health and preventing harm [2].

### Potential Influence of Generational Status

Our sample included first-generation parents who migrated to the Netherlands as well as second generation parents born and raised in the country. As Q-methodology is purely exploratory, we cannot draw conclusions on how generational status would have potentially affected the identified perspectives on HPV vaccination. Nevertheless, prior research on system knowledge of cancer screenings in the Netherlands among first- and second-generation non-Western migrants shows that first-generation individuals have lower system knowledge of the three offered screening programs (breast, cervical and colorectal cancer) compared to native Dutch individuals. Whereas, second-generation individuals have similar levels of knowledge of cervical cancer to native Dutch individuals, but lower system knowledge about breast and colorectal screenings [6]. This suggests that generational status may influence access to health information and healthcare utilization particularly for cervical cancer prevention, which includes HPV vaccination and may therefore, shape attitudes towards HPV vaccination. However, based on our data, we cannot make any definitive statements about this relationship.

### Communalities Across Perspectives

The viewpoints identified in our study are distinct. However, they share some commonalities as presented in Table 3. Parents aligning with Perspectives 1–3 similarly rank the belief that HPV-vaccination is only necessary once individuals become sexually active (st. 5: 4, 5, 5). General information materials on HPV in the Netherlands, provided by the National Institute for Public Health and the Environment (RIVM), state: “You can get HPV (human papillomavirus) through sexual or intimate contact” [54]. This shared source of information may account for the consistency in parental views across perspectives.

Another commonality is that all perspectives agree that the health risks associated with HPV are greater for girls than for boys (st. 12: 5, 5, 6; st. 13: 7, 7, 8). Although parents tend to recognize that HPV is linked to cervical cancer, many appear to be unaware of the implications HPV can have for boys. This may stem from a lack of information and familiarity regarding HPV in males, a gap that remains common among parents [27]. This knowledge gap is likely shaped by traditional public health messaging and media campaigns that have predominantly targeted females [15]. Consequently, females and parents of girls tend to receive stronger and more consistent HPV vaccine recommendations than their male counterparts [26, 39, 46]. The later inclusion of boys in the Dutch National Immunization Program (NIP), which only began in 2022 may have further contributed to this unawareness.

In terms of perceived susceptibility, parents in this study also share similar views regarding the risk of boys contracting HPV, which they were relatively neutral about (st. 2: 5, 4, 5). Interestingly, a few parents mentioned during the interview that boys might be at higher risk of contracting HPV due to having more sexual partners than girls, referencing a gendered double heterosexual standard. We introduced a possible mechanism through which this assumption could occur in the introduction. However, this perception does not emerge as a shared viewpoint in any of the perspectives identified in our study, this highlights a distinction between individual qualitative input and patterns revealed.

### Potential cues to action

The HBM suggests that individuals often require cues to action to take the steps towards particular health behaviour [57], in this case HPV-vaccination for their children. The success of such cues depends on their alignment with parents’ perspectives on HPV-vaccination. The coexistence of multiple perspectives among parents of Turkish and Moroccan origin in the Netherlands calls for tailored strategies to increase HPV-vaccination uptake in these groups.

### Community-based Approach

Information provided by individuals who share similar cultural and religious backgrounds is often perceived as more accessible and easier to process [68]. Parents of Turkish or Moroccan origin may therefore feel more comfortable receiving information from people they know or relate to culturally, religiously or linguistically. In the Netherlands, the National Institute for Public Health and the Environment (RIVM) coordinates the national HPV vaccination programme, while the Municipal Public Health Service (GGD) and, in some regions, the Centre for Youth and Family (CJG) are responsible for its implementation. Across the country, the CJG also provides information to parents about the vaccinations. GGD and CJG efforts could be strengthened by adopting a community-based approach that engages with trusted local community centres, schools or key neighbourhood figures, as they often have established relationships with parents. Such collaborative efforts could enhance understanding, trust, accessibility, and alignment with the specific needs of parents of Turkish or Moroccan origin.

### Creation of properly tailored information

Our findings underscore that insufficient knowledge and uncertainty can be key barriers to HPV vaccination. Cues to action should therefore prioritize clear and accessible information, especially in light of the widespread misinformation surrounding HPV-vaccination [65]. Research suggests that culturally tailored, sensitive approaches are essential for effectively informing vaccine-hesitant individuals across diverse backgrounds, including adolescent females of Turkish and Moroccan descent [24, 58, 66]. As the organization responsible for the national coordination of the vaccination program, RIVM could take the lead in developing clear, culturally sensitive communication materials and guidelines to support local implementation. Locally, GGDs, which implement health programs and conduct community outreach, and CJG professionals, who engage directly with parents and children, can help ensure relevance and accessibility. Parents themselves should also be involved, as they provide the most accurate insight into what information they need and how they prefer to receive it.

### Message framing

Communication should emphasize cancer prevention rather than sexual transmission when discussing HPV-vaccination (cf. [66]). This approach may reduce discomfort, particularly among parents holding the HPV-specific vaccine hesitancy viewpoint and thereby facilitate discussions with their children. In line with effective message framing principles, cues to action should include open dialogue that helps parents recognize the personal relevance of HPV-related risks. When uncertainty is ignored, vaccine messages may appear untrustworthy (Bond & Nolan, 2011,Nyhan et al., 2014). Therefore, strong fear appeals or purely risk-based messages should be avoided unless uncertainties are first addressed, as these can increase refusal among vaccine-hesitant parents (Betsch & Sachse, 2013). Key actors for facilitating this shift include RIVM communication teams, who set the national framework for health messaging, and the GGD and CJG, who translate national messages into locally relevant communication and engage directly with communities.

### Gender inclusivity

Some parents view HPV as a greater threat to girls than boys, reflecting biases in perceived susceptibility and severity. While cervical cancer is a serious consequence for females, HPV also causes cancers in males [52]. Given that many parents in our study are unaware of HPV’s implications for boys, cues to action should address the misconception of HPV only affecting females and emphasize that both genders are vulnerable to HPV-related disease.

### Strengths and Limitations

Using Q-methodology, a technique to explore, understand, and compare different viewpoints [70], our study provided unique insights into the perspectives of parents of Turkish and Moroccan descent regarding HPV-vaccination. One advantage of the Q-method is its flexibility in capturing respondents’ subjective perspectives. However, the method is inductive, and a limitation is that the identified viewpoints are not generalizable to all parents of Turkish and Moroccan descent in the Netherlands. To assess the prevalence of these perspectives within the broader population of Turkish and Moroccan parents in the Netherlands, further quantitative research drawing on population-based samples is needed. Potential ways of doing this could be a Q-based survey design as this allows assessing the prevalence and the antecedents of the stances found in this study [44].

The study included 29 participants, sufficient for Q-methodology. However, the sample’s diversity was somewhat limited. Compared with the Dutch Turkish and Moroccan population, higher-educated parents were, for example, overrepresented and only two respondents had primary or preparatory education Furthermore, most interviews were conducted in the Randstad, a densely populated urban region in the western part of the Netherlands where Turkish and Moroccan communities are concentrated. Note that our study is exploratory and focuses on identifying which perspectives exist, but explicitly not on estimating the prevalence of these perspectives. Therefore, a representative study sample is not required [70]. However, the limited presence of parents with lower levels of education in our sample may have led us to overlook specific perspectives help primarily by parents in these subgroups. Future research could aim for a more educationally diverse sample to capture a broader range of viewpoints.

Respondents’ language preferences may have influenced data collection, which may have introduced bias. Although code-switching and the use of Turkish, Arabic, Berber or Spanish often allowed participants to express themselves more precisely, differences in language proficiency could have affected understanding of statements during the interviews. This was particularly relevant in three interviews: two partially conducted in Berber with assistance from an informal translator, and another in Spanish, as the respondent’s Spanish proficiency exceeded their Dutch, though it was not their mother tongue. Clarifications, peer verification, and informal translators providing in-situ translations mitigated these issues as much as possible, but some cultural or linguistic nuances may nonetheless have been lost or interpreted differently, potentially influencing respondents’ responses. Furthermore, despite the PI’s shared linguistic and cultural background fostering trust, social desirability bias may have affected participants’ answers, particularly on sensitive topics such as sexuality, vaccination decisions and religious practices.

## Conclusion

We identified three distinct viewpoints on HPV-vaccination among parents of Turkish and Moroccan origin using Q-methodology: one characterized by distrust and negative experiences towards vaccination, another shaped by concerns about sexuality and the early age of vaccination, which is specific for HPV immunization, and a third reflecting strong support for immunization. Our findings deepen the understanding of how these parents perceive HPV-vaccination for their children and underscore that vaccine hesitancy is not uniform but varies in reasoning, intensity and focus. By highlighting the heterogeneity in the perspectives on HPV-vaccination held by parents of Turkish and Moroccan, this study contributes important cultural specificity especially because migrant communities and their descendants are often implicitly considered homogenous. This approach moves beyond viewing hesitancy as a simple yes-or-no issue, highlighting how factors like distrust related to the COVID-19 pandemic and pharmaceutical companies, beliefs that HPV-vaccination is not necessary for young children and discomfort discussing sexuality with children interact to shape concerns about vaccine safety and necessity. Moreover, the study adds to existing research by drawing attention to gendered perceptions. Specifically, the underestimation of implications HPV has for boys may make parents less inclined to vaccinate boys. Altogether, these insights emphasize the importance of tailored health promotion strategies that are trust-building, inclusive for all genders, and culturally sensitive. Such insights are crucial for stakeholders and policymakers seeking to increase HPV-vaccination uptake, particularly among populations with the lowest vaccination rates.

## Supplementary Information

Supplementary Material 1.

Supplementary Material 2.

Supplementary Material 3.

Supplementary Material 4.

## Data Availability

The data used in this study are currently unavailable to other researchers, as the research forms part of an ongoing project entitled “Applying a Gender Perspective to Ethnic Differences in HPV-Vaccination Uptake in the Netherlands (GETVACCINL).” The quantitative sorting data will be shared in the EUR data repository under the CC-BY-4.0 license after publication. Qualitative data will not be made available for reuse to safeguard the privacy of our respondents.

## Abbreviations

CBS: Statistics Netherlands (Centraal Bureau voor de Statistiek)
CJG: Centre for Youth and Family (Centrum voor Jeugd en Gezin)
COVID-19: Corona Virus Disease 2019
GGD: Municipal Public Health Services (Gemeentelijke Gezondheidsdiensten)
HBM: Health Belief Model
HPV: Human Papilloma Virus
(H)SDS: (Hetero)Sexual Double Standard
NIP: National Immunization Program
RIVM: National Institute for Public Health and the Environment (Rijksinstituut voor Volksgezondheid en Milleu)
WHO: World Health Organization
