# Supplementary figures and images for: Views on HPV-vaccination held by parents of Turkish and Moroccan origin in the Netherlands: an exploratory study using Q-methodology
Source: BMC Public Health. 2026 Jan 17;26:568. doi: 10.1186/s12889-026-26241-7 (PMC12895906; doi:10.1186/s12889-026-26241-7)

**Supplementary materials**

**1. The 8-item questionnaire used to obtain demographic information**


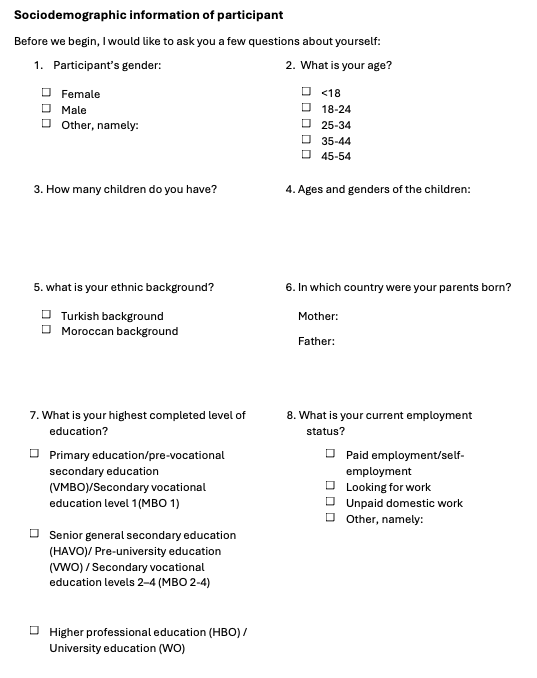

Supplement: Supplementary file 1 — Supplementary Material 1. [file 12889_2026_26241_MOESM1_ESM.docx]
