# Supplementary material for: Views on HPV-vaccination held by parents of Turkish and Moroccan origin in the Netherlands: an exploratory study using Q-methodology
Source: BMC Public Health. 2026 Jan 17;26:568. doi: 10.1186/s12889-026-26241-7 (PMC12895906; doi:10.1186/s12889-026-26241-7)
Supplement: Supplementary file 2 — Supplementary Material 2. [file 12889_2026_26241_MOESM2_ESM.docx]

**Supplementary Material 2:** Detailed background characteristics of participants including additional information on sampling and interview language

| **Participant ID** | **Pseudonym** | **Gender** | **Age category** | **Ethnic Origin (generation)** | **Child(ren)’s gender^a^** | **Age child(ren)^a^** | **Total # of children** | **Education** | **Employment status** | **Sampling method** | **Language of interview** |  |
| --- | --- | --- | --- | --- | --- | --- | --- | --- | --- | --- | --- | --- |
| 1 | Aylin | F | 35-44 | Turkish (II) | F & M | 7 | 2 | Higher education | Employed | IN | NL |  |
| 2 | Burcu | F | 35-44 | Turkish (II) | F & M | 9 & 7 | 2 | Higher education | Employed | SM | NL |  |
| 3 | Kenan | M | 25-34 | Turkish (I) | F | 8 | 2 | Secondary & Vocational education | Employed | Snowball | NL |  |
| 4 | Canan | F | 45-54 | Turkish (I) | M | 8 | 3 | Secondary & Vocational education | Employed | IN | NL |  |
| 5 | Ozan | M | 25-34 | Turkish (II) | F | 7 | 2 | Secondary & Vocational education | Not employed | Snowball | NL |  |
| 6 | Elif | F | 35-44 | Turkish (II) | F | 8 | 2 | Higher education | Employed | IN | NL |  |
| 7 | Karim | M | 35-44 | Moroccan (I) | F | 7 | 3 | Secondary & Vocational education | Employed | IN | NL |  |
| 8 | Selin | F | 45-54 | Turkish (I) | F | 8 | 2 | Secondary & Vocational education | Employed | IN | NL/TR |  |
| 9 | Imane | F | 35-44 | Moroccan (II) | F | 8 | 2 | Secondary & Vocational education | Employed | IN | NL |  |
| 10 | Leyla | F | 35-44 | Turkish (II) | F | 9 | 3 | Secondary & Vocational education | Employed | SM | NL |  |
| 11 | Yasemin | F | 35-44 | Turkish (I) | F | 9 | 3 | Higher education | Employed | IN | TR |  |
| 12 | Sara | F | 35-44 | Moroccan (II) | M | 7 | 3 | Higher education | Employed | CC | NL |  |
| 13 | Rania | F | 45-54 | Moroccan (I) | F | 8 | 2 | Higher education | Not employed | CC | NL |  |
| 14 | Nadia | F | 25-34 | Moroccan (I) | F | 9 | 2 | Secondary & Vocational education | Not employed | CC | NL/BER |  |
| 15 | Samira | F | 35-44 | Moroccan (I) | F | 7 & 8 | 3 | Primary & Preparatory education | Volunteer work | CC | NL/BER |  |
| 16 | Ela | F | 25-34 | Turkish (I) | M | 8 | 2 | Secondary & Vocational education | Employed | IN | NL/TR |  |
| 17 | Idil | F | 35-44 | Turkish (II) | F | 9 | 3 | Higher education | Employed | IN | NL |  |
| 18 | Deniz | M | 25-34 | Turkish (II) | F & M | 7 & 9 | 5 | Higher education | Employed | IN | NL |  |
| 19 | Eren | M | 35-44 | Turkish (II) | F | 8 | 2 | Secondary & Vocational education | Employed | IN | NL |  |
| 20 | Amal | F | 35-44 | Moroccan (II) | M | 8 | 3 | Secondary & Vocational education | Care provider | Street | NL |  |
| 21 | Salma | F | 35-44 | Moroccan (II) | F | 7 | 3 | Higher education | Employed | IN | NL |  |
| 22 | Zahra | F | 45-54 | Moroccan (I) | F | 8 | 2 | Primary & Preparatory education | Not employed | Street | ES//NL |  |
| 23 | Murat | M | 35-44 | Turkish (I) | F | 8 | 2 | Secondary & Vocational education | Employed | IN | TR |  |
| 24 | Amira | F | 35-44 | Moroccan (II) | F | 9 | 3 | Higher education | Employed | Snowball | NL |  |
| 25 | Adam | M | 35-44 | Moroccan (I) | F | 9 | 3 | Secondary & Vocational education | Employed | Snowball | NL |  |
| 26 | Mert | M | 35-44 | Turkish (I) | F | 7 | 1 | Higher education | Employed | Snowball | TR |  |
| 27 | Ata | M | 35-44 | Turkish (I) | F | 9 | 1 | Higher education | Employed | Snowball | TR |  |
| 28 | Naima | F | 25-34 | Moroccan (II) | F | 7 | 4 | Higher education | Employed | IN | NL |  |
| 29 | Baran | M | 25-34 | Turkish (II) | M | 6^b^ | 1 | Secondary & Vocational education | Employed | IN | NL |  |
| *^a^*Only includes children aged 7-9 as that is the inclusion criterion of the study  ^b^1 week until birthday  *Notes:* F = female; M = male; I = 1^st^ generation after migration; II = 2^nd^ generation after migration; III = 3^rd^ generation after migration; IN = informal network; SM = social media; CC = community centre | | | | | | | | | | | | |
