# Supplementary material for: Views on HPV-vaccination held by parents of Turkish and Moroccan origin in the Netherlands: an exploratory study using Q-methodology
Source: BMC Public Health. 2026 Jan 17;26:568. doi: 10.1186/s12889-026-26241-7 (PMC12895906; doi:10.1186/s12889-026-26241-7)
Supplement: Supplementary file 3 — Supplementary Material 3. [file 12889_2026_26241_MOESM3_ESM.docx]

**Supplementary Material 3:** The final statement set including English, Dutch, Turkish and

| Statement | English translation |
| --- | --- |
| 1 | The risk of girls contracting HPV is small. |
| 2 | The risk of boys contracting HPV is small |
| 3 | Girls cannot get HPV because they do not engage in sexual activity before marriage. |
| 4 | Boys cannot get HPV because they do not engage in sexual activity before marriage. |
| 5 | Vaccination against HPV is only necessary when people become sexually active. |
| 6 | Illness and health are in God's hands, so I do not find the HPV vaccine necessary. |
| 7 | It is too early to vaccinate girls against HPV at the age of 10. |
| 8 | It is too early to vaccinate boys against HPV at the age of 10 |
| 9 | Boys and girls can infect each other with HPV, so both should be vaccinated |
| 10 | I do not understand why I should vaccinate my child against HPV |
| 11 | Vaccination against HPV is important to prevent cancer. |
| 12 | HPV is a danger to the health of boys |
| 13 | HPV is a danger to the health of girls |
| 14 | The HPV vaccine protects my child against various types of cancer |
| 15 | Getting vaccinated against HPV has more benefits than drawbacks |
| 16 | I am not vaccinated against HPV myself, so my child does not need it either |
| 17 | The HPV vaccine aligns with my faith because I believe I should take good care of my health |
| 18 | I am against vaccinations in general |
| 19 | The HPV vaccine has harmful side effects for my child |
| 20 | I do not know what the possible side effects of the HPV vaccine are for my child |
| 21 | I do not know enough about the HPV vaccine to decide whether to vaccinate my child |
| 22 | There are substances in the HPV vaccine that are haram. |
| 23 | Vaccinating my child against HPV would harm my family's honour. |
| 24 | I trust the government to ensure that the HPV vaccine is safe. |
| 25 | I trust manufacturers to make a safe HPV vaccine |
| 26 | Vaccinating my child against HPV encourages sexual behaviour |
| 27 | My child receives too many vaccinations at that age |
| 28 | It is a hassle to have my child vaccinated twice a year. |
| 29 | Children should decide for themselves about the HPV vaccine when they are old enough. |
| 30 | The HPV vaccine goes against my culture. |

| Statement | Dutch translation |
| --- | --- |
| 1 | Het risico dat meisjes besmet raken met HPV is klein |
| 2 | Het risico dat jongens besmet raken met HPV is klein |
| 3 | Meisjes kunnen geen HPV krijgen omdat ze geen geslachtsgemeenschap hebben voor het huwelijk |
| 4 | Jongens kunnen geen HPV krijgen omdat ze geen geslachtsgemeenschap hebben voor het huwelijk |
| 5 | Inenten tegen HPV hoeft alleen wanneer mensen seksueel actief worden |
| 6 | Ziekte en gezondheid liggen in Gods handen, dus vind ik de HPV-prik niet nodig |
| 7 | Het is te vroeg om meisjes op 10-jarige leeftijd tegen HPV te laten inenten |
| 8 | Het is te vroeg om jongens op 10-jarige leeftijd tegen HPV te laten inenten |
| 9 | Jongens en meisjes kunnen elkaar besmetten met HPV daarom moeten beiden ingeënt worden |
| 10 | Ik begrijp niet waarom ik mijn kind zou moeten inenten tegen HPV |
| 11 | Inenten tegen HPV is belangrijk om kanker te voorkomen |
| 12 | HPV is een gevaar voor de gezondheid van jongens |
| 13 | HPV is een gevaar voor de gezondheid van meisjes |
| 14 | De HPV-prik beschermt mijn kind tegen verschillende soorten kanker |
| 15 | Je laten inenten tegen HPV heeft meer voordelen dan nadelen |
| 16 | Ik ben zelf niet ingeënt tegen HPV dus mijn kind hoeft dat ook niet |
| 17 | De HPV-prik past bij mijn geloof omdat ik volgens mijn geloof goed voor mijn gezondheid moet zorgen |
| 18 | Ik ben over het algemeen tegen inentingen |
| 19 | De HPV-prik heeft schadelijke bijwerkingen voor mijn kind |
| 20 | Ik weet niet wat de mogelijke bijwerkingen zijn van de HPV-prik voor mijn kind |
| 21 | Ik weet niet genoeg over de HPV-prik om te beslissen of ik mijn kind moet laten inenten |
| 22 | Er stoffen zitten in de HPV-prik die haram zijn |
| 23 | Het inenten van mijn kind tegen HPV zou de eer van mijn familie schaden |
| 24 | Ik vertrouw erop dat de overheid ervoor zorgt dat de HPV-prik veilig is |
| 25 | Ik vertrouw erop dat fabrikanten een veilige HPV-prik maken |
| 26 | Het inenten van mijn kind tegen HPV moedigt seksueel gedrag aan |
| 27 | Mijn kind krijgt op die leeftijd te veel prikken achter elkaar |
| 28 | Het is veel gedoe om mijn kind twee keer per jaar in te laten enten |
| 29 | Kinderen moeten zodra ze oud genoeg zijn zelf beslissen over de HPV-prik |
| 30 | De HPV-prik gaat in tegen mijn cultuur |

| Statement | Turkish translation |
| --- | --- |
| 1 | Kızların HPV'ye yakalanma riski düşüktür |
| 2 | Erkeklerin HPV'ye yakalanma riski düşüktür |
| 3 | Kızlar evlenmeden önce cinsel ilişkiye girmedikleri için HPV kapamazlar |
| 4 | Erkekler evlenmeden önce cinsel ilişkiye girmedikleri için HPV kapamazlar |
| 5 | HPV aşısı yalnızca insanlar cinsel olarak aktif hale geldiklerinde yapılmalıdır |
| 6 | Hastalık ve sağlık Allah'tan gelir, bu yüzden HPV aşısına gerek duymuyorum |
| 7 | Kızları 10 yaşındayken HPV’ye karşı aşılamak çok erken |
| 8 | Erkekleri 10 yaşındayken HPV’ye karşı aşılamak çok erken |
| 9 | Erkekler ve kızlar birbirlerine HPV bulaştırabilirler, bu nedenle her ikisinin de aşılanması gerekmektedir |
| 10 | Çocuğumu neden HPV'ye karşı aşılatmam gerektiğini anlamıyorum |
| 11 | HPV'ye karşı aşılanmak kanseri önlemek için önemlidir |
| 12 | HPV, erkeklerin sağlığı için bir tehdittir |
| 13 | HPV, kızların sağlığı için bir tehdittir |
| 14 | HPV aşısı, çocuğumu çeşitli kanser türlerine karşı korur |
| 15 | HPV'ye karşı aşı olmanın faydaları dezavantajlarından daha fazladır |
| 16 | Kendim HPV’ye karşı aşılanmadım, o yüzden çocuğum için de gerek bulmuyorum |
| 17 | HPV aşısı inancıma uygun çünkü inancım sağlığımı korumam gerektiğini belirtiyor |
| 18 | Genel olarak aşı karşıtıyım |
| 19 | HPV aşısının çocuğum için zararlı yan etkileri var |
| 20 | Çocuğum için olan HPV aşısının olası yan etkilerini bilmiyorum |
| 21 | HPV aşısı hakkında yeterince bilgiye sahip olmadığım için çocuğumu aşılatıp aşılatmamak konusunda karar veremiyorum |
| 22 | HPV aşısında haram olan maddeler bulunuyor |
| 23 | Çocuğumu HPV'ye karşı aşılatmak ailemizin onurunu zedeleyebilir |
| 24 | Devletin, HPV aşısının güvenli olmasını sağlayacağına inanıyorum |
| 25 | Üreticilerin güvenli bir HPV aşısını ürettiklerine inanıyorum |
| 26 | Çocuğumu HPV'ye karşı aşılamak cinsel davranışı teşvik ediyor |
| 27 | Çocuğum o yaşta art arda çok fazla aşı oluyor |
| 28 | Çocuğuma bir yılda iki kez aşı yaptırmak çok uğraştırıyor |
| 29 | Çocuklar yeterince büyüdüklerinde HPV aşısı hakkında kendileri karar vermelidir |
| 30 | HPV aşısı kültürüme aykırı |

| Statement | Arabic translation |
| --- | --- |
| 1 | خطر إصابة الفتيات بفيروس الورم الحليمي البشري ضئيل |
| 2 | إن خطر إصابة الأولاد بفيروس الورم الحليمي البشري منخفض |
| 3 | لا يمكن أن تصاب الفتيات بفيروس الورم الحليمي البشري لأنهن لا يمارسن الجنس قبل الزواج |
| 4 | لا يمكن للأولاد أن يصابوا بفيروس الورم الحليمي البشري لأنهم لا يمارسون الجنس قبل الزواج |
| 5 | التطعيم ضد فيروس الورم الحليمي البشري ضروري فقط عندما يصبح الشخص نشطًا جنسيًا |
| 6 | المرض والصحة بيد الله، لذلك لا أعتقد أن حقنة فيروس الورم الحليمي البشري ضرورية |
| 7 | من السابق لأوانه تطعيم الفتيات ضد فيروس الورم الحليمي البشري في سن العاشرة |
| 8 | من السابق لأوانه تطعيم الأولاد ضد فيروس الورم الحليمي البشري في سن العاشرة |
| 9 | يمكن للفتيان والفتيات أن ينقلوا العدوى لبعضهم البعض بفيروس الورم الحليمي البشري، لذا يجب تطعيم كليهما |
| 10 | لا أفهم لماذا يجب علي تطعيم طفلي ضد فيروس الورم الحليمي البشري |
| 11 | التطعيم ضد فيروس الورم الحليمي البشري مهم للوقاية من السرطان |
| 12 | يشكل فيروس الورم الحليمي البشري خطراً على صحة الأولاد |
| 13 | فيروس الورم الحليمي البشري يشكل خطرا على صحة الفتيات |
| 14 | لقاح فيروس الورم الحليمي البشري يحمي طفلي من أنواع مختلفة من السرطان |
| 15 | للتطعيم ضد فيروس الورم الحليمي البشري مزايا أكثر من عيوبه |
| 16 | لم يتم تطعيمي ضد فيروس الورم الحليمي البشري بنفسي، لذا لا يحتاج طفلي إلى أن يتم تطعيمه أيضًا |
| 17 | يتناسب تطعيم فيروس الورم الحليمي البشري مع إيماني لأنه وفقًا لإيماني يجب أن أعتني بصحتي جيدًا |
| 18 | أنا بشكل عام ضد التطعيمات |
| 19 | لحقن فيروس الورم الحليمي البشري آثار جانبية ضارة على طفلي |
| 20 | لا أعرف ما هي الآثار الجانبية المحتملة للتطعيم ضد فيروس الورم الحليمي البشري على طفلي |
| 21 | لا أعرف ما يكفي عن لقاح فيروس الورم الحليمي البشري لأقرر ما إذا كان ينبغي عليّ تطعيم طفلي |
| 22 | تحتوي حقنة فيروس الورم الحليمي البشري على مواد محرمة |
| 23 | إن تطعيم طفلي ضد فيروس الورم الحليمي البشري من شأنه أن يلحق الضرر بشرف عائلتي |
| 24 | أنا على ثقة من أن الحكومة ستضمن أن التطعيم ضد فيروس الورم الحليمي البشري آمن |
| 25 | أنا على ثقة من أن الشركات المصنعة تصنع حقنة آمنة لفيروس الورم الحليمي البشري |
| 26 | تطعيم طفلي ضد فيروس الورم الحليمي البشري يشجع السلوك الجنسي |
| 27 | يحصل طفلي على عدد كبير جدًا من الحقن المتتالية في هذا العمر |
| 28 | إن تطعيم طفلي مرتين في السنة أمر صعب للغاية |
| 29 | يجب على الأطفال أن يقرروا بأنفسهم التطعيم ضد فيروس الورم الحليمي البشري بمجرد أن يبلغوا السن الكافي |
| 30 | إن لقاح فيروس الورم الحليمي البشري يتعارض مع ثقافتي |
