# Supplementary material for: Views on HPV-vaccination held by parents of Turkish and Moroccan origin in the Netherlands: an exploratory study using Q-methodology
Source: BMC Public Health. 2026 Jan 17;26:568. doi: 10.1186/s12889-026-26241-7 (PMC12895906; doi:10.1186/s12889-026-26241-7)
Supplement: Supplementary file 4 — Supplementary Material 4. [file 12889_2026_26241_MOESM4_ESM.docx]

**Table S3:** Rotated factor loadings of participants on the extracted perspectives

| **Participant ID** | **Perspective 1** | **Perspective 2** | **Perspective 3** | **Uniqueness** |
| --- | --- | --- | --- | --- |
| 1 | 0.1341 | 0.6791 | -0.2746 | 0.4454 |
| 2 | -0.2184 | 0.0468 | 0.8250 | 0.2696 |
| 3 | 0.5881 | 0.2922 | 0.3089 | 0.4734 |
| 4 | 0.2393 | 0.5289 | 0.1633 | 0.6363 |
| 5 | 0.4270 | 0.5904 | -0.0508 | 0.4666 |
| 6 | 0.1921 | -0.0543 | 0.5533 | 0.6541 |
| 7 | 0.6523 | 0.2839 | -0.2795 | 0.4158 |
| 8 | 0.3164 | 0.0028 | 0.4235 | 0.7205 |
| 9 | 0.6351 | 0.1627 | -0.1858 | 0.5357 |
| 10 | 0.5580 | 0.3823 | 0.4194 | 0.3666 |
| 11 | 0.1017 | 0.7308 | 0.0253 | 0.4550 |
| 12 | 0.6564 | 0.2346 | 0.0075 | 0.5140 |
| 13 | 0.6487 | -0.1134 | 0.2932 | 0.4803 |
| 14 | 0.7202 | 0.2291 | -0.0785 | 0.4227 |
| 15 | 0.7558 | -0.0107 | -0.2498 | 0.3663 |
| 16 | -0.3783 | -0.1700 | 0.5542 | 0.5209 |
| 17 | 0.5070 | 0.2885 | 0.2271 | 0.6081 |
| 18 | 0.4661 | 0.0686 | -0.2512 | 0.7150 |
| 19 | 0.7114 | 0.2042 | 0.3463 | 0.3323 |
| 20 | 0.8296 | 0.4115 | 0.1562 | 0.1181 |
| 21 | 0.1758 | -0.0821 | 0.5825 | 0.6231 |
| 22 | -0.0936 | -0.1721 | 0.5822 | 0.6227 |
| 23 | -0.0568 | 0.1297 | 0.9442 | 0.0884 |
| 24 | 0.1219 | 0.5401 | 0.1174 | 0.6796 |
| 25 | 0.1899 | 0.4580 | 0.0175 | 0.7538 |
| 26 | -0.0762 | 0.2494 | 0.8066 | 0.2814 |
| 27 | -0.0392 | 0.0998 | 0.8982 | 0.1818 |
| 28 | -0.0738 | 0.4335 | 0.7431 | 0.2544 |
| 29 | 0.5712 | 0.3419 | -0.2588 | 0.4899 |
| **Note.** Values represent rotated factor loadings indicating the strength of association between each participant’s responses and the extracted perspectives. Higher absolute values reflect stronger alignment with a given perspective. Uniqueness indicates the proportion of variance in a participant’s responses not explained by the extracted factors (i.e., unexplained variance). Participants were assigned to the perspective on which they loaded most stronglt above the predefined threshold (±0.36). | | | | |
